# TNF reduces osteogenic cell fate in PDL cells at transcriptional and functional levels without alteration of periodontal proliferative capacity

**DOI:** 10.1007/s00056-024-00541-2

**Published:** 2024-08-02

**Authors:** Isabel Knaup, Rafael Kramann, Martha-Julia Sasula, Paula Mack, Rogério Bastos Craveiro, Christian Niederau, Franziska Coenen, Sabine Neuss, Joachim Jankowski, Michael Wolf

**Affiliations:** 1 Department of Orthodontics, Medical Faculty, RWTH Aachen University, Pauwelsstr. 30, 52074 Aachen, Germany; 2 Clinic for Renal and Hypertensive Disorders, Rheumatological and Immunological Diseases (Medical Clinic II), Medical Faculty, RWTH Aachen University, Aachen, Germany; 3 Department of Gastroenterology, Hepatology and Transplant Medicine, Medical Faculty, University of Duisburg-Essen, Essen, Germany; 4 Helmholtz Institute for Biomedical Engineering, BioInterface Group, RWTH Aachen University, Aachen, Germany; 5 Institute of Pathology, Medical Faculty, RWTH Aachen University, Aachen, Germany; 6 Institute for Molecular Cardiovascular Research, Medical Faculty, RWTH Aachen University, Aachen, Germany

**Keywords:** Orthodontic tooth movement, Periodontal ligament, PDL fibroblasts, Tumor necrosis factor, Osteogenic differentiation, Kieferorthopädische Zahnbewegung, Parodontalligament, PDL-Fibroblasten, Tumornekrosefaktor, Osteogene Differenzierung

## Abstract

**Aims:**

To investigate the effect of tumor necrosis factor (TNF) on the growth of human periodontal ligament (PDL) cells, their osteogenic differentiation and modulation of their matrix secretion in vitro.

**Methods:**

The influence of 10 ng/ml TNF on proliferation and metabolic activity of PDL cells was analyzed by cell counting (DAPI [4’,6-diamidino-2-phenylindole] staining) and the MTS (3-(4,5-dimethylthiazol-2-yl)-5-(3-carboxymethoxyphenyl)-2-(4-sulfophenyl)-2H-tetrazolium) assay. In addition, cells were cultured under control conditions and osteogenic conditions (media containing 10 mM β-glycerophosphate). Quantitative expression analysis of genes encoding the osteogenic markers alkaline phosphatase (*ALP)*, collagen type I alpha 1 chain (*COL1A1*), osteoprotegerin (*OPG*), and osteopontin (*OPN*) was performed after 7 and 14 days of cultivation. Calcium deposits were stained with alizarin red.

**Results:**

Our studies showed that 10 ng/ml TNF did not affect the survival and metabolic activity of PDL cells. Quantitative expression analysis revealed that long-term cultures with TNF impaired osteogenic cell fate at early and late developmental stages. Furthermore, TNF significantly reduced matrix secretion in PDL cells.

**Conclusion:**

The present data confirm TNF as a regulatory factor of proinflammatory remodeling that influences the differentiation behavior but not the metabolism and cell proliferation of the periodontium. Therefore, TNF represents an interesting target for the regulation of orthodontic remodeling processes in the periodontium.

## Abstract (de)

**Ziele:**

In-vitro-Untersuchung des Einflusses von Tumornekrosefaktor (TNF) auf das Wachstum humaner PDL(Parodontalligament)-Zellen, ihre osteogene Differenzierung und die Modulation ihrer Matrixsekretion.

**Methoden:**

Der Einfluss von 10 ng/ml TNF auf Proliferation und metabolische Aktivität der PDL-Zellen wurde anhand der Zellzählung (DAPI [4ʼ,6-Diamidino-2-Phenylindol]-Färbung) und des MTS (3-(4,5-Dimethylthiazol-2-yl)-5-(3-Carboxymethoxyphenyl)-2-(4-Sulfophenyl)-2H-Tetrazolium) Assays analysiert. Darüber hinaus wurden die Zellen unter Kontrollbedingungen und osteogenen Bedingungen (Medien mit 10 mM β‑Glycerophosphat) kultiviert. Eine quantitative Expressionsanalyse von Genen, die für die osteogenen Marker alkalische Phosphatase (ALP), Kollagen-Typ-I-Alpha-1-Kette (COL1A1), Osteoprotegerin (OPG) und Osteopontin (OPN) kodieren, erfolgte nach 7 und 14 Tagen Kultivierung. Kalziumablagerungen wurden mit Alizarinrot angefärbt.

**Ergebnisse:**

Unsere Untersuchungen zeigten, dass 10 ng/ml TNF keinen Einfluss auf das Überleben und die Stoffwechselaktivität von PDL-Zellen hatte. Quantitative Expressionsanalysen ergaben, dass Langzeitkulturen mit TNF das osteogene Zellverhalten in frühen und späten Entwicklungsstadien beeinträchtigten. Darüber hinaus ist die Matrixsekretion von TNF-stimulierten PDL-Zellen signifikant reduziert.

**Schlussfolgerungen:**

Die vorliegenden Daten bestätigen TNF als regulatorischen Faktor des proinflammatorischen Remodelings, welcher das Differenzierungsverhalten, aber nicht den Metabolismus und die Zellproliferation des Parodonts beeinflusst. TNF stellt somit ein interessantes Target zur Modulation kieferorthopädischer Umbauprozesse im Zahnhalteapparat dar.

## Introduction

Orthodontic tooth movement (OTM) is induced by the application of active mechanical forces that induce a local noninfectious inflammatory response in the periodontal ligament (PDL). Mechanosensory cells, which include PDL fibroblasts, cementoblasts, bone mesenchymal stem cells (BMSCs), periodontal ligament stem cells (PDLSCs), osteoblasts, osteocytes, and osteoclasts, sense the strain caused by these external forces and respond by deforming themselves or the extracellular matrix (ECM) [1]. This process is accompanied by the synthesis and secretion of various mediators that lead to bone resorption and deposition, as well as remodeling of the PDL [1].

**Fig. 1:**
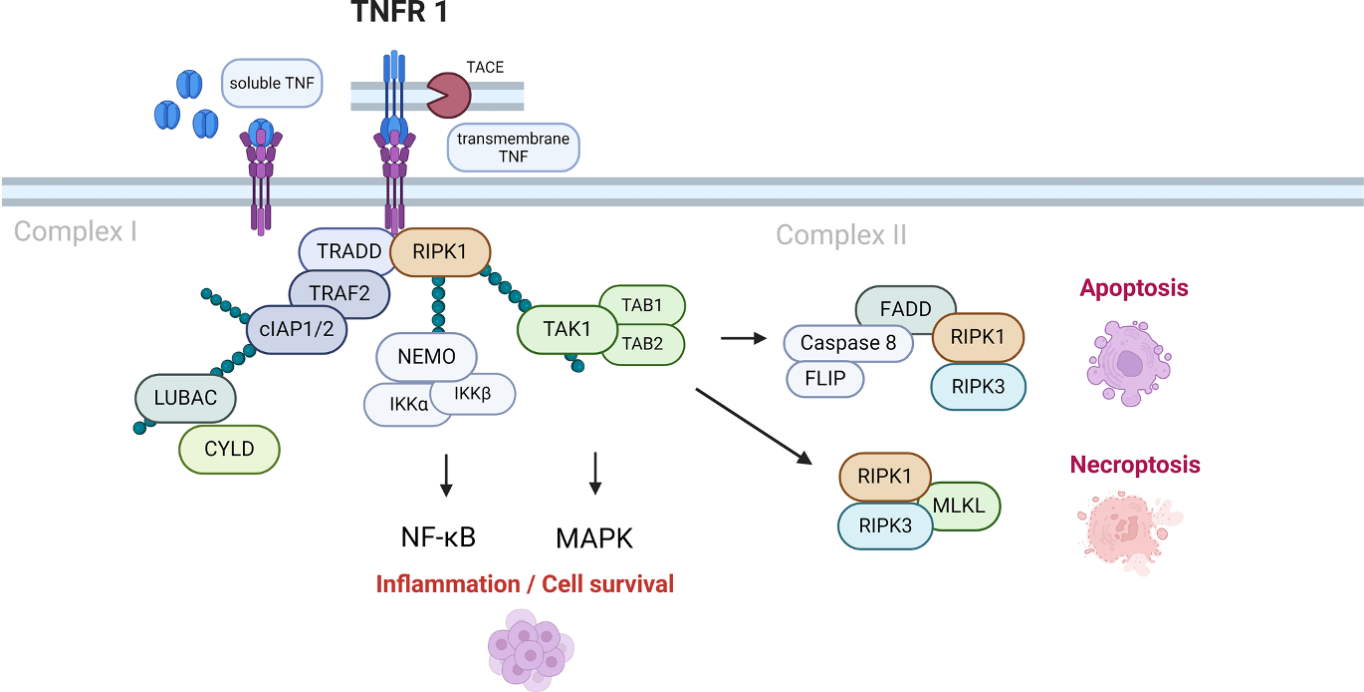
Tumor necrosis factor receptor 1 (TNFR1) signaling. Schematic illustration of TNFR1 signaling after Schlicher et al. [11]; illustration adapted from “TNF Pathway”, by BioRender.com (2022). Retrieved from https://app.biorender.com/biorender-templates TNFR1(Tumor necrosis factor receptor 1)-Signalweg. Schematische Darstellung des TNFR1-Signalwegs in Anlehnung an Schlicher et al. [11]; Illustration adaptiert von „TNF Pathway“ auf BioRender.com (2022). Abgerufen von https://app.biorender.com/biorender-templates

PDL cells are the most abundant cells in PDL tissue and are thought to be the first to respond to mechanical stress [1]. PDL cells have been shown to contain mesenchymal progenitor cells with multilineage differentiation potential comparable to that of bone marrow-derived mesenchymal stem cells (MSCs) [2–4]. Thus, PDL cells exhibit an osteogenic phenotype and contribute to the regeneration and remodeling processes of alveolar bone, cementum, and PDL during OTM [5–7]. The differentiation of PDL cells is tightly regulated by complex cytokine networks such as TNF. The effects of TNF are essentially mediated by binding to two distinct receptors, tumor necrosis factor receptor 1 (TNFR1) and tumor necrosis factor receptor 2 (TNFR2), with most activities being signaled by TNFR1 [8, 9]. When TNF binds to TNFR1, TNF receptor-associated death domain protein (TRADD) is activated, which can induce the activation of signaling pathways with a leading role in controlling cell survival and inflammation ([10, 11]; Fig. 1).

In the context of inflammatory conditions, such as OTM, the generation of inflammatory stimuli, including IL12, IFN‑γ, and ROS, by damaged tissue promotes the polarization of macrophages into the M1 phenotype [12]. This process results in the release of cytokines, such as TNF, IL6, IL1, and others, which affect osteoclast differentiation and formation [12]. Two mechanisms have been identified by which TNF promotes osteoclastogenesis: directly, by increasing the population and/or differentiation of osteoclast precursors; and indirectly, by enhancing the secretion of RANKL by osteoblasts and other cells [12]. Moreover, TNF has been demonstrated to be capable of inducing a transformation in CD11b ^+^ F4/80^+^ cells (bone marrow cells) from Ly6C^−^Gr1^−^M2 macrophages to Ly6C^−^Gr1^−^CD11c^+^- and Ly6C^+^Gr1^−^ CD11c ^+^ M1 macrophages [12]. This may act as a boost of osteoclastic precursors to M1 macrophages, with elevated osteoclastogenesis potentials [12]. Studies have shown that TNF-dependent TNFR1 signaling is involved in periapical bone resorption [15] and plays a role in osteoclast and odontoclast formation during OTM [14]. Consistent with this, Jäger et al. showed that systemic application of soluble receptors to TNF reduced the amount of root resorption and odontoclasts in rats, but also the amount of tooth movement and osteoclasts [8]. In addition, TNF appears to affect the vascular network by inducing the expression of vascular endothelial growth factor (VEGF), which plays a key role in the chemotaxis of osteoclast precursors during OTM [16].

TNF also reduces osteoblast differentiation by inhibiting RUNX2, AP‑1, and SATB2 [17–25], and induces osteoblast apoptosis [1, 26, 27]. In addition, TNF has been shown to inhibit bone morphogenetic protein (BMP) signaling and subsequent bone formation [28, 29]. Interestingly, Yao et al. demonstrated a strong upregulation of TNF in rats during tooth eruption with subsequent upregulation of BMP‑2 and BMP‑3 at the base of the tooth crypt, suggesting largely unknown functions of TNF during tooth development [30].

However, the effect of TNF on PDL cells, which are considered to be bone progenitor cells, is not fully understood, although TNF has been widely studied in promoting inflammation and osteoclastogenesis. In the present work, we stimulated PDL cells with TNF to investigate possible effects on cell survival and analyzed early and late osteogenic differentiation markers to shed light on possible regulative effects on periodontal tissue regeneration and the compelling role of TNF during periodontal tissue remodeling.

## Materials and methods

### Cell culture

Human periodontal ligament fibroblasts (HPDLF CC-7049, Lonza, Basel, Switzerland) were cultured in Dulbecco’s modified Eagle’s medium (DMEM; 4.5 g/L glucose) containing 10% FCS, 50 mg/L ascorbic acid, 100 u/ml penicillin, 100 µg/ml streptomycin at 37 °C, 5% CO_2_, and 95% humidity. When cells reached confluence, they were passaged with 0.05% trypsin/EDTA. All experiments were performed with cells in passage two.

### Proliferation assay (DAPI staining)

To analyze proliferation capacity 1000 cells were cultured in 6‑well plates for 6, 24, 48, 72, and 96 h and challenged with or without 10 ng/ml recombinant human TNF (Thermo Fisher Scientific, Carlsbad, CA, USA) based on recent literature [31]. Cells were fixed with 4% PFA (paraformaldehyde) for 10 min and washed three times with PBS (phosphate buffered saline) containing 0.1% Triton™ X‑100 (Merck Millipore, Burlington, MA, USA). DAPI (4’,6-diamidino-2-phenylindole) staining (Thermo Fisher Scientific, Carlsbad, CA, USA; 1:10,000 in PBS) was applied for 10 min.

### MTS assay

The MTS assay was performed at 4000 cells/well in 96-well plates. Cells were cultured in 50 µl of medium (DMEM, Thermo Fisher Scientific, Carlsbad, CA, USA) containing 1% FCS. After 24 h, 10 ng/ml TNF was added. Cell proliferation assays were performed after 6, 24, 48, and 72 h using 20 µl CellTiter 96® Aqueous One Solution containing 3‑(4,5-dimethylthiazol-2-yl)-5-(3-carboxymethoxyphenyl)-2-(4-sulfophenyl)-2H-tetrazolium (MTS; Promega, Madison, WI, USA). After incubation for 2 h at 37 °C, the absorbance was measured at 490 nm using an ELISA plate reader (infinite m nano, Tecan, Männedorf, Switzerland). Cell viability was calculated relative to the untreated control.

### Relative expression of osteogenic markers

The relative gene expression of the osteogenic markers alkaline phosphatase (*ALP*), collagen type I alpha 1 chain (*COL1A1*), osteoprotegerin (*OPG*), osteopontin (*OPN*) was performed as follows: 4 × 10^5^ cells were seeded in 6‑well plates and cultured under control (culture medium; see above) or mineralizing conditions (culture medium +10 mM β-glycerophosphate) with or without 10 ng/ml TNF (Thermo Fisher Scientific, Carlsbad, CA, USA). After 7 and 14 days, cells were harvested using TRIzol™ reagent (Invitrogen, Thermo Fisher Scientific, Carlsbad, CA, USA) for RNA isolation. For RNA isolation, cells were harvested after 7 and 14 days using TRIzol™ reagent and BCP (1-bromo-3-chloropropane; 1:10). Samples were centrifuged (12,000 g; 15 min; 4 °C) for phase separation. The aqueous phase was transferred to a microcentrifuge microtube. RNA was precipitated with 2‑propanol, washed with ethanol (75%), and dissolved in H_2_O. After RNA purification according to the manufacturer’s instructions for the Quick-RNA™ Microprep Kit (Zymo Research, Freiburg, Germany), including an on-column DNA digestion, RNA purification, and concentration were verified photometrically by analyzing A260/A280 and A260/A230 ratios (NanoDrop One™, Thermo Fisher Scientific, Carlsbad, CA, USA). RNA was reverse transcribed into cDNA using oligo(dT)18 primers and SuperScript III RT (Invitrogen, Thermo Fisher Scientific, Carlsbad, CA, USA). Quantitative RT-PCR was performed in triplicates using 25 ng/µl cDNA, 0.5 µM primers (eurofins; self-designed, intron-spanning, Luxembourg) and Luminaris Color HiGreen qPCR Master Mix (Thermo Fisher Scientific, Carlsbad, CA, USA). The PCR reaction was run for a total of 40 cycles with a subsequent melting curve for PCR product analysis in a qTower3 (Analytik Jena, Jena, Germany). Gene expression was normalized to ribosomal protein L22 (*RPL22*). Primer sequences are shown in Table 1**.**

**Table 1:** qPCR primer sequences indicated in 5’–3’ direction for human gene targets qPCR-Primersequenzen in 5’–3’-Richtung für humane Zielgene

Gene symbol	Gene name	Accession number (NCBI GenBank)	Primer sequence	Product size (bp)
*ALP*	Alkaline phosphatase	NM_000478.6	fw GACCTCGTTGACACCTGGAA	190
			rev CCACCATCTCGGAGAGTGAC	
*COL1A1*	Collagen type I	NM_000088.4	fw CAACAGCCGCTTCACCTACA	79
	alpha 1 chain		rev TTCAATCACTGTCTTGCCCCA	
*OPG*	Osteoprotegerin	NM_002546.4	fw GCAACACAGCTCACAAGAACA	142
			rev GTTAGCATGTCCAATGTGCCG	
*OPN*	Osteopontin	NM_000582.3	fw TGATTTTCCCACGGACCTGC	186
			rev TCGCTTTCCATGTGTGAGGT	
*RPL22*	Ribosomal protein L22	NM_000983.4	fw TGATTGCACCCACCCTGTAG	98
			rev GGTTCCCAGCTTTTCCGTTC	

*NCBI* National Center for Biotechnology Information; *fw* forward; *rev* reverse; *bp* base pair, *qPCR* quantitative polymerase chain reaction

### Osteogenesis assay

For the osteogenesis assay, 10,000 cells/well were seeded in 24-well plates and cultured under control (culture medium; see above) or osteogenic conditions (culture medium +10 mM β-glycerophosphate) and challenged with or without 10 ng/ml TNF (Thermo Fisher Scientific, Carlsbad, CA, USA) based on previous studies [5, 32, 33]. After 7 and 14 days, cells were fixed in ice-cold ethanol (70%) for 1 h at room temperature. Cells were carefully washed three times with ddH_2_O and stained with alizarin red solution (40 mM) for 10 min. Cells were then gently washed three times with PBS for 5 min on a rotating shaker and photographed. For further quantification, cells were lysed with acetic acid (10%) for 30 min, scraped from the well and transferred to a microcentrifuge tube. Samples were heated at 85 °C for 10 min, cooled on ice for 5 min, and centrifuged (13,000 g, 5 min). After neutralization with ammonium hydroxide (10%; 1:2), alizarin red staining was quantified by measuring optical density at 405 nm using a microplate reader (Infinite M Nano; Tecan).

### Microscopy, image analysis, and statistics

**Fig. 2:**
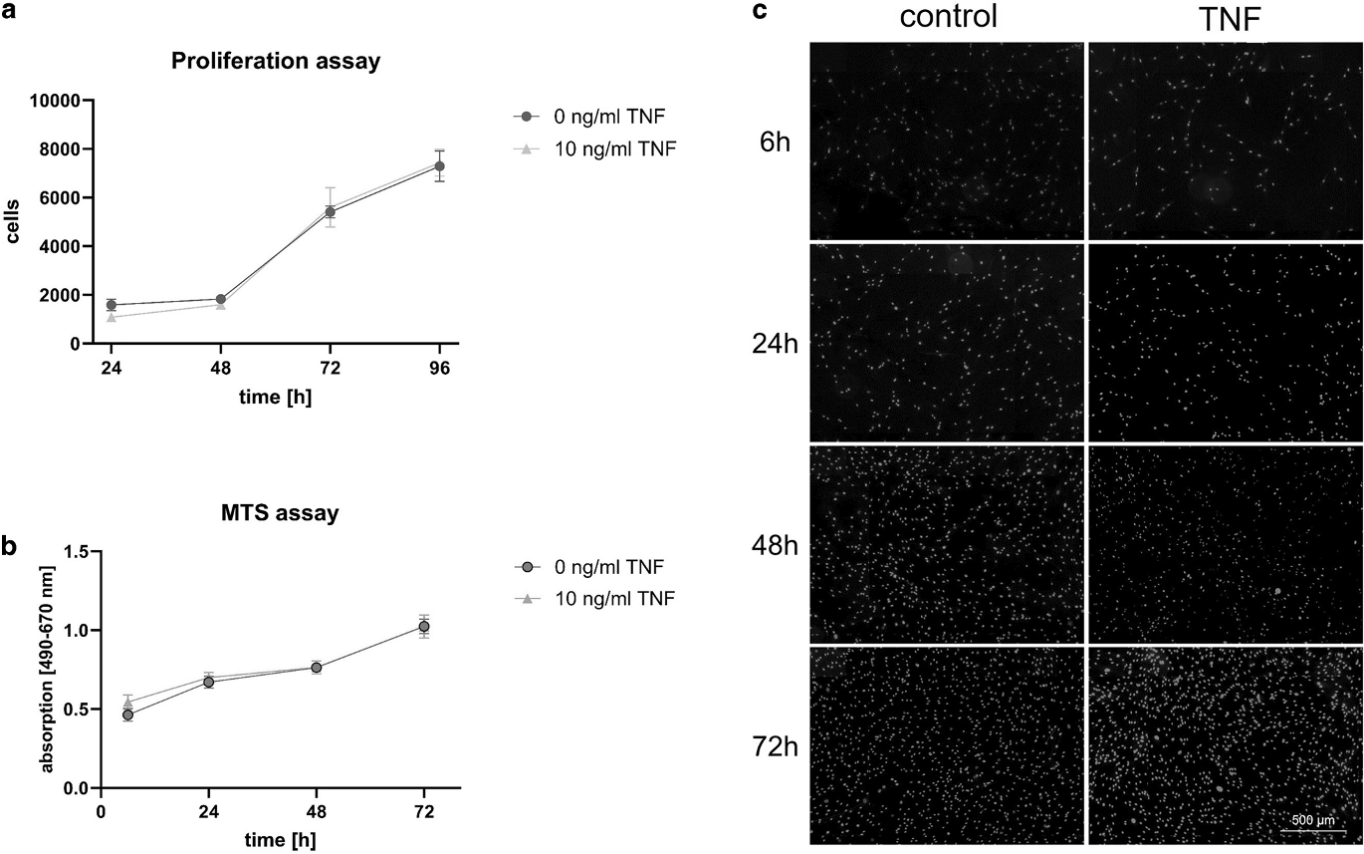
Tumor necrosis factor (TNF) stimulation does not reduce regenerative capacity of human periodontal ligament (PDL) cells. Proliferation (**a**) and metabolism (**b**) assay of PDL cells treated with 10 ng/ml compared to control for 24, 48, 72, or 96 h. **c** Cell nuclei were stained with DAPI (*white*) TNF (Tumornekrosefaktor) beeinflusst die Regenerationsfähigkeit humaner PDL(Parodontalligament)-Zellen nicht. Proliferations- (**a**) und metabolisches Assay (**b**) humaner PDL-Zellen, die im Vergleich zur Kontrolle über 24, 48, 72 oder 96 h mit 10 ng/ml TNF stimuliert wurden. **c** Zellkerne wurden mit DAPI (*weiß*) gefärbt

**Fig. 3:**
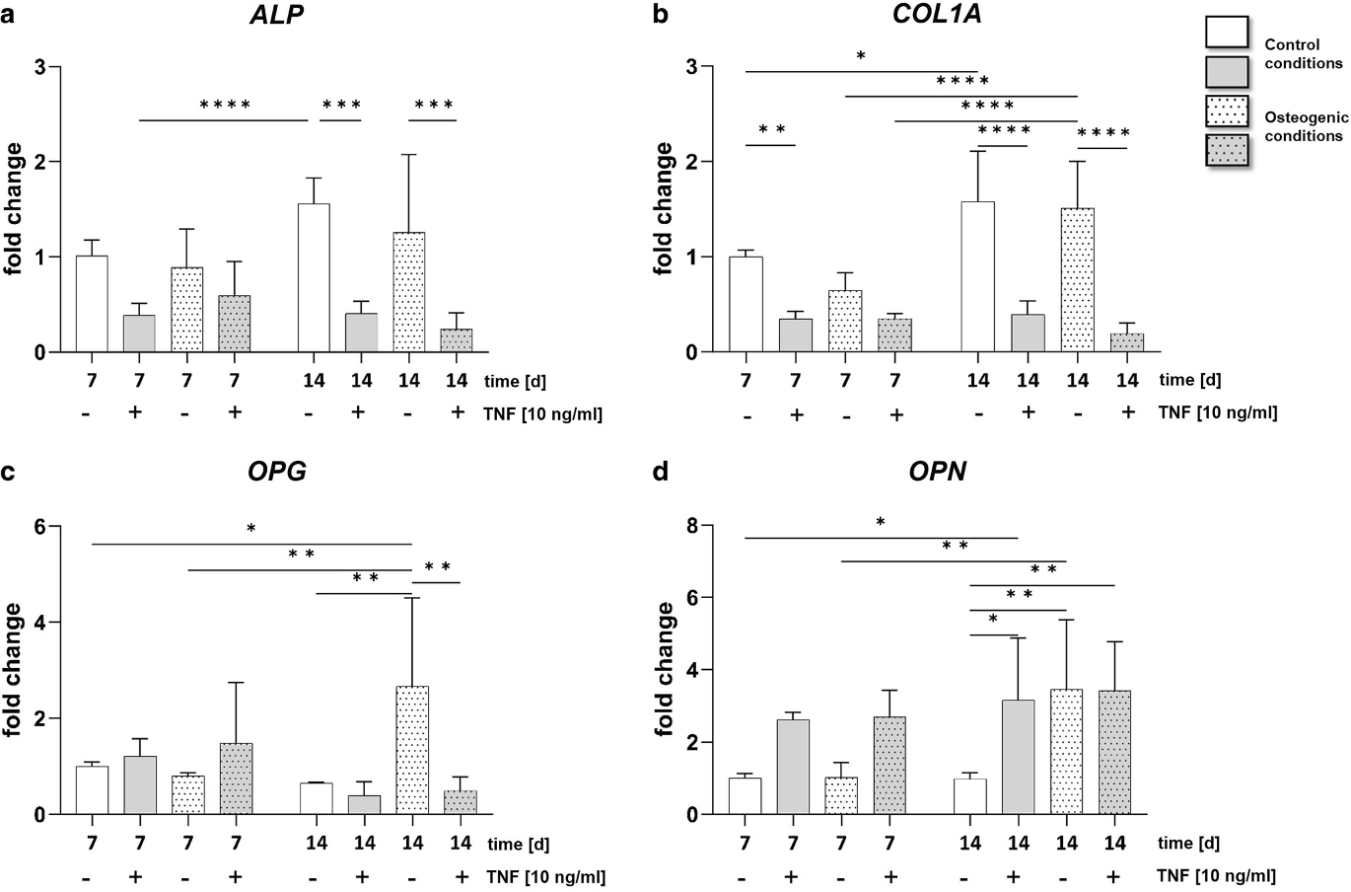
Tumor necrosis factor (TNF) reduces osteogenic cell fate in human periodontal ligament (PDL) cells. Quantitative expression analysis of genes encoding the osteogenic markers (**a**) alkaline phosphatase (*ALP*), (**b**) collagen type I alpha 1 chain (*COL1A1*), and (**c**) osteoprotegerin (*OPG*) as well as for the osteoclast activating marker (**d**) osteopontin (*OPN*) in PDL cells cultured with 10 ng/ml TNF for 7 or 14 days under control (*white *and* gray bars*) and osteogenic conditions (*white *and* gray patterned bars*). Data are normalized to the expression levels of the reference gene *RPL22* and displayed relative to the control condition; *d* days; *P < 0.05, **P < 0.01, ***P < 0.001, ****P < 0.0001 TNF (Tumornekrosefaktor) reduziert die frühe und späte osteogene Differenzierung von PDL(Parodontalligament)-Zellen. Quantitative Expressionsanalysen von Genen, die für die osteogenen Marker (**a**) alkalische Phosphatase (*ALP*), (**b**) Kollagen-Typ-I-Alpha-1-Kette (*COL1A1*) und (**c**) Osteoprotegerin (*OPG*) sowie für den osteoklastenaktivierenden Marker (**d**) Osteopontin (*OPN*) kodieren von PDL-Zellen, die über 7 oder 14 Tage mit 10 ng/ml TNF unter Kontroll- (*weiße *und* graue Balken*) und osteogenen Bedingungen (*weiße *und* graue gemusterte Balken*) kultiviert wurden. Die Daten werden auf die Expressionsniveaus der Referenzgene *RPL22* normalisiert und auf die Kontrolle normiert; *d* Tage; *p < 0,05, **p < 0,01, ***p < 0,001, ****p < 0,0001

**Fig. 4:**
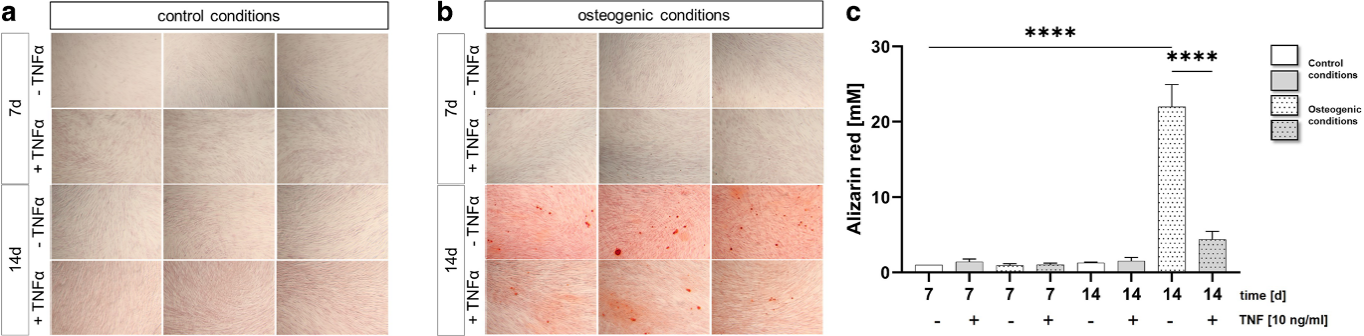
Tumor necrosis factor (TNF) reduces the mineralization potential of human periodontal ligament (PDL) cells. **a**, **b** Mineralization assay of PDL cells cultured with 10 ng/ml TNF for 7 or 14 days under control (**a**) and osteogenic conditions (**b**). Calcium deposits were stained with alizarin red and shown as representative sections. **c** Plot of alizarin red quantification results. *d* days; ****P < 0.0001 TNF verringert das Mineralisierungspotenzial humaner PDL(Parodontalligament)-Zellen. **a** /**b** Mineralisierungsassay von PDL-Zellen, die über 7 oder 14 Tage mit 10 ng/ml TNF unter Kontroll- (**a**) und osteogenen Bedingungen (**b**) kultiviert wurden. Kalziumablagerungen wurden mit Alizarinrot angefärbt und als repräsentative Ausschnitte dargestellt. **c** Ergebnisse der Alizarinrot-Quantifizierung. *d* Tage; ****p < 0,0001

Images were acquired with an inverted confocal laser scanning microscope (Zeiss AxioVision, Carl Zeiss, Jena, Germany) and analyzed with Affinity Designer 2 (Serif, West Bridgford, UK). Figure 1 was created using BioRender.com (BioRender, Toronto, ON, Canada). The illustrations in Figs. 2, 3, and 4 were generated using GraphPad Prism (version 9, GraphPad Software, San Diego, CA, USA). The Kolmogorov–Smirnov test was used to assess normality. Mean ± standard deviation (SD) is presented unless otherwise noted. One-way analysis of variance (ANOVA) was used for statistical analysis. All analyses were performed at the 5% significance level. Experiments were repeated twice and performed in triplicate.

## Results

### TNF maintains proliferative capacity and metabolic activity in human PDL cells

To investigate the effects of TNF on the survival of human PDL cells, a proliferation assay was performed. The cell number and percentage of proliferative cells were analyzed at each time point for control and experimental conditions (Fig. 2a, c). The results showed that PDL cells proliferated slowly in linear growth on days 1–2 under both the experimental and control conditions. On days 3–4, cell proliferation was in logarithmic growth phase and PDL cells showed relatively strong proliferative capacity. However, no statistically significant differences were observed between the two conditions (*p* ≥ 0.05).

To analyze possible toxic effects of TNF on human PDL cells, an MTS assay was performed over 3 days (Fig. 2b). The results showed that the absorbance, and metabolic activity of PDL cells increased in linear growth proportional to the cell proliferation on days 1–3 in both control and experimental conditions and no cytotoxic effects were observed. No statistically significant differences between the conditions analyzed were documented (*p* ≥ 0.05).

### Long-term culture with TNF impairs osteogenic cell fate in PDL cells

PDL cells have been reported to have osteogenic differentiation potential [4]. To determine the effects of TNF on osteogenesis in PDL cells, quantitative PCR was performed for genes encoding the osteogenic markers collagen type I alpha 1 chain (*COL1A1*), alkaline phosphatase (*ALP*), osteoprotegerin (*OPG*), and osteopontin (*OPN*).

The results showed that treatment with 10 ng/ml TNF significantly decreased the gene expression of *ALP* and *COL1A1* on day 14 (*ALP p* ≤ 0.001; *COL1A1 p* ≤ 0.0001; Fig. 3a, b). These effects were observed under both control and osteogenic conditions. In addition, 14 days of TNF treatment reduced the expression of *OPG* in PDL cells under osteogenic conditions (*p* ≤ 0.01; Fig. 3c). In contrast, gene expression of *OPN*, a chemotactic marker for osteoclast precursors, was significantly upregulated in cells stimulated with TNF on day 14 under control conditions (*p* ≤ 0.01; Fig. 3d). Quantitative expression analysis revealed that prolonged cultivation with 10 ng/ml TNF impaired osteogenic cell fate.

### Matrix maturation and mineralization is decreased by TNF stimulation in PDL cells

To analyze the effect of TNF on matrix maturation and mineralization, an alizarin red staining of the PDL cells was performed. Cells were cultured under control (Fig. 4a) and osteogenic conditions (Fig. 4b) and stimulated with 10 ng/ml TNF for 14 days. The results showed that the cells reached a multilayer stage under both conditions on day 7 under both conditions, but no mineralized deposits were detectable. On day 14, induction of osteogenesis was observed and alizarin red staining identified a significant amount of calcium deposits in PDL cells under osteogenic conditions. PDL cells under control conditions did not exhibit calcium deposits. Interestingly, 10 ng/ml TNF led to a significant reduction in calcification in PDL cells under mineralizing conditions (Fig. 4c) and, therefore, appears to induce changes not only at the transcriptional level but also at the functional level.

## Discussion

PDL cells play a pivotal role in the remodeling of the alveolar bone and the connective tissues in response to mechanical stimuli applied during orthodontic tooth movement [34, 35]. As PDL cells share similarities with bone progenitor cells [5–7], their differentiation is tightly regulated by complex cytokine networks [1]. Changes in the functionality of PDL cells, such as those induced by excessive activation of TNF signaling under inflammatory conditions, may affect the quality of the tooth attachment and the capacity for tooth movement [36]. To detect possible toxic effects of TNF in PDL cells, we performed proliferation and MTS assays and demonstrated that TNF did not affect the survival and metabolic activity of PDL cells. Based on this, TNF seems to not affect the regenerative potential of PDL cells, which is characterized by multipotency, high proliferative capacity, and the potential to regenerate bone, cementum, and PDL tissue [37].

During OTM, TNF promotes bone resorption and reduces bone formation. Liu et al. have recently demonstrated in rats that the M1-type macrophage immune microenvironment in particular promoted activities associated with epithelial–mesenchymal transition, fiber degradation, osteoclastogenesis, and inflammation through the Wnt, IL-17, and TNF signaling pathways, whereas the M2-type macrophage immune microenvironment showed superiority in inducing epithelial induction, fiber formation, and mineralization performance of PDLCs by upregulating the TGFβ and PI3K-Akt signaling pathways [38]. Lin et al. reviewed the pathogenesis of noninfectious inflammatory root resorption and found out that elevation of M1-type macrophages and TNF levels played a pivotal role in activating clastic cells during OTM but also in root resorption processes [39]. TNF blockers have been successfully developed and used in the clinical treatment of autoimmune disorders. However, recent approaches to systemically inhibit TNF by administration of the biologic infliximab, reduced root resorption but also tooth movement in rats [40]. In addition, serious side effects such as infections, malignancies, and autoimmune diseases are discussed as being associated with TNF blockers [9].

The present study investigated the possible effects of TNF on early and late osteogenic differentiation. Our results showed that TNF significantly decreased the gene expression of *COL1A1, ALP,* and *OPG* in PDL cells. COL1A1 and ALP are relevant markers of extracellular matrix secretion during the second developmental stage of osteoblasts after osteoblastic lineage commitment and active proliferation. Here, immature osteoblasts differentiate into mature osteoblasts that secrete COL1A1 as a major component of the extracellular matrix and express ALP to mature the extracellular matrix [25, 41–43]. Based on these results, TNF significantly seems to reduce early and late osteogenic differentiation. To confirm our hypothesis, we performed a mineralization assay in PDL cells that were challenged with TNF and demonstrated significantly reduced calcium deposition. Thus, TNF appears to induce changes not only at the transcriptional level but also at the functional level.

PDL cells orchestrate bone and periodontal remodeling by regulating the differentiation and function of bone-resorbing osteoclasts through the production of receptor activator of nuclear factor-kappa B ligand (RANKL) and macrophage colony-stimulating factor (M-CSF) [44, 45]. Binding of RANKL and M‑CSF to the RANK and cFMS receptors (Colony-stimulating factor 1 receptors), respectively, on the surface of osteoclast precursors, induces enhanced osteoclast differentiation, proliferation, and survival [13, 25]. In addition, PDL cells secrete OPG, a key negative regulator of osteoclastogenesis that inhibits the terminal stages of osteoclast differentiation, suppresses matrix osteoclast activation, and competes with RANK for RANKL binding, thereby, accelerating osteoclast apoptosis [44, 45]. Our results showed that TNF significantly reduced *OPG* and, thus, promoted osteoclast differentiation.

To further evaluate the effects of TNF on osteoclast differentiation, we determined the gene expression of *OPN* in challenged PDL cells. Our results showed that TNF significantly upregulated *OPN* gene expression in the short and long term, whereas it was highly upregulated under mineralizing conditions independent of TNF. OPN is a multifunctional protein that contributes to bone remodeling, e.g., by promoting osteoclastogenesis, osteoclast activity, survival, and motility [46]. Knockout studies have shown that bone remodeling was impaired in OPN-deficient mice when subjected to mechanical stress [46–50]. In the early stages of orthodontic tooth movement, OPN was observed in osteocytes, whereas in later stages, OPN was ubiquitously expressed in PDL cells, osteoclasts, cementocytes, cementoblasts, and osteoblasts, as well as in the cement line of alveolar bone and cementum [46, 47, 51, 52].

However, OPN has been shown to inhibit the mineralization of osteoblast cultures in a phosphate-dependent manner [46]. Furthermore, OPN was highly induced during inflammatory activation and rapidly upregulated by TNF [46, 53]. Taken together, our results confirm *OPN* as a relevant marker in periodontal remodeling that is upregulated under inflammatory conditions. The observed upregulation of *OPN* under mineralizing conditions may be part of a negative feedback mechanism to avoid excessive mineralization.

There are several limitations to the presented findings. First, the commercial cells used in this study were only superficially characterized, and their representativeness may be limited to the context presented. Second, we focused on several differentiation markers that we considered representative based on previous studies. Investigation of additional markers at different time points may provide clearer insights into the effects of TNF on periodontal remodeling and its potential as a target for the regulation of orthodontic processes. Finally, the results must be interpreted with caution because they reflect an in vitro approach and are theoretical in nature.

Thus, our data provide evidence that TNF stimulates PDL cell proliferation in the short term, but prolonged culture reduces the osteogenic phenotype.

## Conclusions

Based on our findings, we summarize:

- Elevated tumor necrosis factor (TNF) levels did not decrease the metabolic activity or proliferation of periodontal ligament (PDL) cells. Thus, the regenerative capacity of PDL cells was not altered by TNF.
- TNF decreased the osteogenic cell fate in PDL cells by reducing gene expression of early and late osteogenic markers, promoting osteoclastogenesis and increasing matrix secretion. TNF appears to induce changes not only at the transcriptional level but also at the functional level.

The present data confirm TNF as a regulatory factor of proinflammatory remodeling that affects the differentiation behavior but not the metabolism and cell proliferation of periodontal stromal cells. Clinically, TNF antagonism may be of interest in patients with reduced periodontal attachment or chronic inflammation. Further studies are needed to fully elucidate the effects of TNF in this context and to provide clinical implications.
